# Evaluation of adverse drug reaction formatting in drug information databases

**DOI:** 10.5195/jmla.2020.983

**Published:** 2020-10-01

**Authors:** Sean M. McConachie, Derek Volgyi, Hannah Moore, Christopher A. Giuliano

**Affiliations:** 1 et6398@wayne.edu, Eugene Applebaum College of Pharmacy and Health Sciences, Wayne State University, Detroit, MI, and, Beaumont Hospital, Dearborn, MI; 2 d.volgyi@wayne.edu, Eugene Applebaum College of Pharmacy and Health Sciences, Wayne State University, Detroit, MI; 3 fo8589@wayne.edu, Eugene Applebaum College of Pharmacy and Health Sciences, Wayne State University, Detroit, MI; 4 ek2397@wayne.edu, Eugene Applebaum College of Pharmacy and Health Sciences, Wayne State University, Detroit, MI, and, Ascension St. John Hospital, Dearborn, MI

## Abstract

**Objective:**

The research evaluated the differences in formatting of adverse drug reaction (ADR) information in drug monographs in commonly used drug information (DI) databases.

**Methods:**

A cross-sectional analysis of formatting of ADR information for twenty commonly prescribed oral medications in seven commonly used DI databases was performed. Databases were assessed for presentation of ADR information, including presence of placebo comparisons, severity of ADR, onset of ADR, formatting of ADRs in percentile (quantitative) format or qualitative format, whether references were used to cite information, whether ADRs are grouped by organ system, and word count of the ADR section. Data were collected by two study investigators and discrepancies were resolved via consensus. Chi-square analyses and one-way analysis of variance (ANOVA) were used to evaluate for mean group differences in categorical and continuous data, respectively.

**Results:**

The seven DI databases varied significantly on each analyzed ADR variable, including variables known to impact interpretation such as placebo comparisons and qualitative versus quantitative formatting. Placebo comparisons were most common among monographs in Micromedex In-Depth Answers (70%) but were absent among monographs in Epocrates, Lexicomp, and Micromedex. Quantitative information was commonly used in most databases but was absent in Epocrates. Average word counts were higher in Clinical Pharmacology and Micromedex In-Depth answers compared to other databases.

**Conclusion:**

Substantial variation in ADR formatting exists between the most common DI databases. These differences may translate into alternative interpretations of medical information and, thus, impact clinical judgment. Further studies are needed to assess whether these differences impact clinical practice.

## INTRODUCTION

Adverse drug reactions (ADRs), which are noxious and unintended responses to medication at normal doses, contribute substantially to patient morbidity and mortality [1–3]. Recent estimates suggest that ADRs may occur in over 16% of hospitalized patients, and the Food and Drug Administration (FDA) reports that medication errors may injure over a million people annually [2, 4]. Health librarians and clinicians alike are well positioned to prevent and reduce the impact of ADRs in clinical practice by identifying and acting on unbiased medication information. Recent research suggests that clinical pharmacy interventions can reduce the number of ADRs and drug-related problems in both hospitalized and ambulatory patients [5–7].

Health librarians, physicians, pharmacists, and other clinicians now rely on drug information (DI) databases to quickly access drug monograph information to help with clinical decision making [8]. One situation in which DI databases may be referenced is in differentiating a medication-induced ADR from an adverse drug event. An adverse drug event is an untoward medical occurrence during treatment that is not caused by the medication itself [3]. Unfortunately, the many available DI databases vary substantially as to the specific information that they present and their utility in answering different types of clinical questions [9–11].

Recent research suggests that differences in the presentation of medication information may impact interpretation of the risks and benefits of therapy [12–14]. For example, it is known that both physicians and patients are influenced by the way that risk and benefit information related to medications is displayed. Patient interpretation of a medication's risks can be influenced by the presence or absence of corresponding placebo information when ADR information is displayed [15].

Likewise, both patient and physician perceptions of medication efficacy differ when presented with relative risk or absolute risk information [16]. There is even evidence to suggest that perceptual differences may impact clinical decision making [12, 17–19]. Most recently, a study demonstrated that pharmacists and pharmacy students were more likely to deem an ADR to be medication-induced if they were given information derived from Micromedex: In-Depth Answers, compared to participants who received ADR information derived from Lexicomp or Epocrates [17].

Given the possible consequences of deciding to discontinue or continue a potentially ADR-inducing medication, it is important for DI databases to present this information in a manner that is easy to interpret. Additionally, it is important for clinicians and librarians to gain insight into how these databases are formatted so that they can quickly identify the necessary information from a database that utilizes a format that is optimized for interpretation and sound decision making. The objective of this study was to evaluate differences in the formatting of ADR information in drug monographs in commonly used DI databases.

## METHODS

This cross-sectional study evaluated the presentation of ADR information contained in drug monographs in commonly used DI databases. Seven electronic DI resources were selected for analysis: Micromedex®, Micromedex: In-Depth Answers, Epocrates®, Lexicomp®, Clinical Pharmacology®, RxList.com, and Physician's Desk References (via pdr.net). For the purposes of this study, the adverse events section of Micromedex and the additional ADR information contained in Micromedex: In-Depth Answers were considered separately, because their ADR formatting differed substantially, even though the two were linked within a single database. Database analysis was performed during the period of July 1, 2019, to August 30, 2019.

To gain a representative sample of medication monographs, the twenty most highly prescribed prescription medications in the United States, as defined by the Medication Expenditure Panel Survey, were selected to be searched individually in each DI database [20]. A full list of the assessed medications and databases is in Table 1.

**Table 1:** Evaluated medications and drug information databases

Top 20 oral medications in United States	Drug information databases
Levothyroxine	Micromedex
Lisinopril	Micromedex: In-Depth Answers
Atorvastatin	
Metformin	Epocrates
Amlodipine	Lexicomp
Metoprolol tartrate	Clinical Pharmacology
Omeprazole	RxList.com
Simvastatin	PDR (pdr.net)
Losartan	
Albuterol	
Gabapentin	
Hydrochlorothiazide	
Acetaminophen-hydrocodone	
Sertraline	
Furosemide	
Fluticasone	
Acetaminophen	
Amoxicillin	
Alprazolam	
Atenolol	

Presented in order of use as of August 2019.

Each ADR section in the monographs was evaluated for the following formatting criteria: qualitative (use of words) or quantitative (use of numbers) formatting of ADR frequency, presence or absence of comparative placebo frequency, severity assessment for ADRs, onset information for ADRs, grouping of ADRs by organ system, presence or absence of references for ADR information, and total word count of the ADR section. These factors were selected based on formatting requirements listed in prevailing FDA guidance documents for ADR section labeling of package inserts (frequency, severity, onset, comparator information), and other criteria were selected based on consensus discussions of the investigative team (word count, references, qualitative, and quantitative information) [21]. The investigative team only analyzed the formatting of drug information and did not analyze specific content related to the twenty medications. Following assessment of all medication monographs in each individual DI database, the investigators assessed the percentage of the medications that matched the criterion for each category for the cumulative database.

Two investigators individually (Moore and Volgyi) assessed medication monographs for each of the DI databases. Discrepancies were resolved via consensus and input from a third investigator (McConachie or Giuliano). Chi-square analysis and one-way analysis of variance (ANOVA) were used to evaluate for between-group differences in categorical and continuous variables, respectively. An a priori alpha value of 0.05 was established.

## RESULTS

Every medication (n=20) contained a representative monograph in each of the DI databases, allowing complete comparison for each of the formatting variables (Table 2). There were statistically significant differences (*p*0.01 for all comparisons) among DI databases for each of the analyzed ADR variables. Every database, except for Epocrates, utilized quantitative formatting for ADR prevalence. Quantitative formatting was most common in Micromedex In-Depth Answers (100%) and completely absent in Epocrates (0). Most monographs also framed the ADRs qualitatively, for example, using terminology such as “common” or “serious.” Epocrates, Micromedex, and Clinical Pharmacology used qualitative wording in each of the monographs (100%), whereas Lexicomp did not use qualitative wording in any of the monographs (0). There was also a large discrepancy in whether databases included comparative placebo information (comparative frame) in the ADR sections or whether ADR information for the medication was stated alone (single frame). Only Micromedex: In-Depth Answers monographs used the comparative frame in the majority of its monographs (75%). Comparative frames were also used in Clinical Pharmacology and RxList.com (30% for both) but were absent from all monographs from Micromedex, Lexicomp, and Epocrates (0).

**Table 2:** Adverse drug reaction formatting differences between databases

Database adverse drug reaction (ADR) variable	Micro-medex	Micromedex: In-Depth Answers	Epocrates	Lexicomp	Clinical Pharmacology	RxList. com	Physician's Desk Reference	Statistical significance	
Quantitative formatting	85%	100%	0	80%	95%	75%	90%	χ^2^(6)=74.7	*p*<0.01
Qualitative formatting	100%	95%	100%	0	100%	85%	0	χ^2^(6)=123.8	*p*<0.01
Placebo comparison	0	75%	0	0	30%	30%	0	χ^2^(6)=82.9	*p*<0.01
ADR onset	0	100%	0	0	75%	15%	95%	χ^2^(6)=110.0	*p*<0.01
Grouping by organ system	100%	100%	0	100%	90%	90%	0	χ^2^(6)=123.3	*p*<0.01
Severity assessment	100%	100%	100%	0	5%	55%	5%	χ^2^(6)=102.0	*p*<0.01
References provided	0	100%	0	20%	100%	55%	0	χ^2^(6)=105.8	*p*<0.01
Word count: mean (± SD)	77 (± 37)	6,883 (± 3,942)	50 (± 18)	234 (± 117)	1,660 (± 712)	663 (± 400)	462 (± 184)	F (6,133)=49.8	*p*<0.01

Onset information for ADRs was commonly included in Micromedex: In-Depth Answers (100%) and PDR (95%) but was absent from Micromedex, Lexicomp, and Epocrates monographs (0). The majority of monographs grouped ADRs by organ system; however, Epocrates and PDR did not. Severity assessment was included for all monographs in Micromedex, Micromedex: In-Depth Answers, and Epocrates databases but was completely absent from Lexicomp. DI databases also varied widely in whether specific references were provided for ADR information. References were included in all monographs in Clinical Pharmacology and Micromedex: In-Depth Answers but was absent in monographs from Micromedex, Epocrates, and PDR. Finally, databases were significantly different with respect to the average word count of the ADR section of the monograph. On the upper end, Micromedex: In-Depth Answers utilized the most words (6,883 words), followed by Clinical Pharmacology (1,660 words), and all other monographs were less than 700 words in length.

## DISCUSSION

This study demonstrates the significant variability in the formatting of ADR information currently contained in DI databases. Every ADR formatting factor in the study demonstrated significant variation among available databases including those known to impact clinical decision making, such as frequency formatting and placebo comparison rates. These prevalent formatting differences have the potential to influence clinical practice, because surveys demonstrate that a majority of pharmacists reference DI databases on a daily basis or more frequently [17]. Additionally, it is known that a substantial proportion of DI questions posed in practice deal with potential ADRs. By responding to such queries, pharmacists and other clinicians can add value to pharmacovigilance in health care settings [8, 22–24].

Unlike other DI questions, which can be fairly straightforward (such as those concerning dosing, contraindications, or administration instructions), ADR questions are unique because they often require an assessment of causality, which is subject to interpretation [25]. The numerous scoring systems designed to assess ADR probability in practice demonstrate a large amount of disagreement, as do experts when they are asked to assess likely ADR causality [26–29]. The ambiguity surrounding ADR interpretation creates a situation in which clinical judgment is more likely to be required.

However, it is well established that judgment of medication information is influenced by the formatting of the medication risk and efficacy information [12–14]. For example, when given comparative efficacy information in terms of relative risk, physicians and patients are both far more likely to view the drug as favorable than when given the same information in an absolute risk format [16]. Additionally, patients are more likely to view a drug as risky when given comparative placebo information than when the drug is presented with medication risk information alone [15]. Intuitively, providing medication information in a comparative format is more likely to lead to a more informed medical decision. In fact, surveys demonstrate that the majority of physicians feel that pharmaceutical companies should be required to provide medication risk and efficacy information alongside corresponding placebo information [15]. It is surprising, therefore, that the vast majority of DI databases provided medication ADR rates in a single-frame format. In fact, only Micromedex: In-Depth Answers provided this information for the majority of the analyzed drug monographs. Health librarians could use this information to guide students, clinicians, and patients toward databases that provide comparative formatting.

Other formatting parameters can also impact clinical decision making [30, 31]. For example, health communication researchers often advocate for the use of quantitative rather than qualitative formatting of frequencies because quantitative formatting gives a more detailed assessment of risk [32]. However, Epocrates, a widely used DI database, contains no quantitative risk information, instead labels ADR frequencies under ambiguous headings such as “common.” Additional formatting variables that were determined to be significantly different between databases in this study—including word count, grouping of reactions by organ system, or inclusion of onset and severity information—are not well studied in the risk communication literature. It is unknown whether differences between these parameters could impact clinical decision making or whether clinicians have a particular preference for one format over another. Further studies in this area are needed as this could impact which databases health librarians recommend to clinicians in practice.

The variability of both content and formatting of ADRs in DI resources is alarming given that the resources are designed to improve clinical decision making. This variation raises the question of whether librarians, pharmacists, and other medical practitioners should have more of a say in how these databases are constructed. There is no literature that the authors are aware of that has sought to achieve consensus among medical practitioners as to how to optimize the clinical utility of DI databases for physicians, pharmacists, or other health care providers. This is likely the only method to determine whether formatting differences in word count, grouping mechanism, and referencing impact users or are likely to impact patient safety and care. Until that point, database users should be wary of the limitations and biases of individual databases and consider referencing multiple DI sources in practice.

This study has a number of limitations. First of all, the analysis was limited in scope. Only twenty medications were used as a representative sample for all prescription medications that are currently approved for use. However, by evaluating the most commonly used medications, including a mix of medications approved both before and after the Food, Drug, and Cosmetic Act and the Kefauver-Harris Amendments (the passage of which changed regulations regarding the quantity of clinical safety and efficacy studies that were required prior to drug approval), the authors believe the information drawn from the databases was likely representative of medication monographs, in general [33]. Additionally, the study lacks an assessment of user preference. Currently, the optimal method of formatting ADR information in DI databases is unknown, and there is no professional guidance in this area. Other studies that have evaluated user preference for DI databases have generated mixed results [10, 24].

In the absence of clinical outcome studies or professional consensus, it is impossible to estimate the optimal method of formatting ADR information in regard to criteria such as word count, referencing, grouping of ADRs, and framing of ADR information. Finally, the study did not evaluate other factors, such as content or ease of use, which may influence the choice to use one particular DI database over another. However, this study does provide a starting point upon which further discussion and future studies regarding the impact of ADR formatting on clinical decision making can be built.

Despite these limitations, this study provides meaningful data demonstrating significant and potentially clinical meaningful differences among current DI databases. It also demonstrates that there is substantial variation in DI databases regarding the content and formatting of ADR information. As there is evidence suggesting that information formatting can impact risk interpretation and subsequent clinical decision making, librarians and clinicians must be wary of where and how they are finding and interpreting DI in clinical practice.

## References

[1] Kongkaew C, Noyce PR, Ashcroft DM Hospital admissions associated with adverse drug reactions: a systematic review of prospective observational studies. Ann Pharmacother. 2008 7;42(7):1017–25. 18594048 10.1345/aph.1L037

[2] Miguel A, Azevedo LF, Araujo M, Pereira AC Frequency of adverse drug reactions in hospitalized patients: a systematic review and meta–analysis. Pharmacoepidemiol Drug Saf. 2012 11;21(11):1139–54. DOI: 10.1002/pds.3309. 22761169

[3] World Health Organization (WHO). WHO draft guidelines for adverse event reporting and learning systems Geneva, Switzerland: The Organization; 2005.

[4] Institute for Safe Medication Practices. Baxter and the Institute for Safe Medication Practices (ISMP) address global medication error prevention [Internet] The Institute; 2017 [cited 21 May 2019]. <https://www.ismp.org/news/baxter-and-institute-safe-medication-practices-ismp-address-global-medication-error-prevention>.

[5] Dempsey JT, Matta LS, Carter DM, Stevens CA, Stevenson LW, Desai AS, Cheng JW Assessment of drug therapy-related issues in an outpatient heart failure population and the potential impact of pharmacist-driven intervention. J Pharm Pract. 2017 6;30(3):318–23. DOI: 10.1177/0897190016641491. 27080398

[6] Hamblin S, Rumbaugh K, Miller R Prevention of adverse drug events and cost savings associated with PharmD interventions in an academic level I trauma center: an evidence–based approach. J Trauma Acute Care Surg. 2012 12;73(6):1484–90. DOI: 10.1097/TA.0b013e318267cd80. 23064610

[7] Schnipper JL, Kirwin JL, Cotugno MC, Wahlstrom SA, Brown BA, Tarvin E, Kachalia A, Horng M, Roy CL, McKean SC, Bates DW Role of pharmacist counseling in preventing adverse drug reactions after hospitalization. Arch Int Med. 2006 3 13;166(5):565–71. 16534045 10.1001/archinte.166.5.565

[8] Belgado BS, Hatton RC, Doering PL Evaluation of electronic drug information resources for answering questions received by decentralized pharmacists. Am J Health-Syst Pharm. 1997 11 15;54(22):2592–6. 9397221 10.1093/ajhp/54.22.2592

[9] Clauson KA, Marsh WA, Polen HH, Seamon MJ, Ortiz BI Clinical decision support tools: analysis of online drug information databases. BMC Med Inform Decis Mak. 2007 3 8;7:7 DOI: 10.1186/1472-6947-7-7. 17346336 PMC1831469

[10] Moutford CM, Lee T, De Lemos J, Loewen PS Quality and usability of common drug information databases. Can J Hosp Pharm. 2010 3;63(2):130–7. 22478968 10.4212/cjhp.v63i2.898 PMC2858502

[11] Rambaran KA, Huynh HA, Zhang Z, Robles J The gap in electronic drug information resources: a systematic review. Cureus. 2018 6 22;10(6):e2860 DOI: 10.7759/cureus.2860. 30148013 PMC6107040

[12] Bui TC, Krieger HA, Blumenthal-Barby JA Framing effects on physicians' judgement and decision making. Psychol Rep. 2015 10;117(2):508–22. DOI: 10.2466/13.PR0.117c20z0. 26444837

[13] Gong J, Zhang Y, Yang Z, Huang Y, Feng J, Zhang W The framing effect in medical decision-making: a review of the literature. Psychol Health Med. 2013;18(6):645–53. DOI: 10.1080/13548506.2013.766352. 23387993

[14] McGettigan P, Sly K, O'Connell D, Hill S, Henry D The effects of information framing on the practices of physicians. J Gen Intern Med. 1999 10;14(10):633–42. 10571710 10.1046/j.1525-1497.1999.09038.x PMC1496755

[15] O'Donohughe AC, Sullivan HW, Aikin KJ Randomized study of placebo and framing information in direct-to-consumer print advertisements for prescription drugs. Ann Behav Med. 2014 12;48(3):311–22. DOI: 10.1007/s12160-014-9603-1. 24682975 PMC7370304

[16] Perneger TV, Agoritsas T Doctors and patients' susceptibility to framing bias: a randomized trial. J Gen Intern Med. 2011 12;26(12):1411–7. DOI: 10.1007/s11606-011-1810-x. 21792695 PMC3235613

[17] McConachie SM, Giuliano CA, Mohammad I, Kale-Pradhan PB Adverse drug reactions in drug information databases: does presentation affect interpretation? J Med Libr Assoc. 2020 1;108(1):76–83. DOI: 10.5195/jmla.2020.748. 31897054 PMC6919994

[18] McNeil BJ, Pauker SG, Sox HC Jr., Tversky A On the elicitation of preferences for alternative therapies. N Engl J Med. 1982 5 27;306(21):1259–62. DOI: 10.1056/nejm198205273062103. 7070445

[19] O'Connor AM, Boyd NF, Tritchler DL, Kriukov Y, Sutherland H, Till JE Eliciting preferences for alternative cancer drug treatments. the influence of framing, medium, and rater variables. Med Decis Making. 1985 Winter;5(4):453–63. DOI: 10.1177/0272989x8500500408. 3842425

[20] Medical Expenditure Panel Survey (MEPS). The top 200 of 2020: provided by the ClinCalc DrugStats Database [Internet] Version 20.0. Rockville, MD: Agency for Healthcare Research and Quality (AHRQ); 2007–2017 [cited 1 Aug 2019]. <https://clincalc.com/DrugStats/Top200Drugs.aspx>.

[21] Food Drug Administration Center for Drugs Evaluation Research. Guidance for industry: adverse reactions section of labeling for human prescription drug and biological products—content and format [Internet] The Administration; 2006 [cited 12 May 2020]. <https://www.fda.gov/media/72139/download>.

[22] Perez-Ricart A, Gea-Rodriguez E, Roca-Montanana A, Gil-Manez E, Perez-Feliu A Integrating pharmacovigilance into the routine of pharmacy department: experience of nine years. Farm Hosp. 2019 7 1;43(4):128–33. DOI: 10.7399/fh.11169. 31276445

[23] Leufkens HG Pharmacy-led pharmacovigilance: ready for use or missed opportunity? Pharmacoepidemiol Drug Saf. 2019 12;28(12):1562 DOI: 10.1002/pds.4901. 31714644

[24] Carvajal MJ, Clauson KA, Gershman J, Polen HH Associations of gender and age groups on the knowledge and use of drug information resources by American pharmacists. Pharm Pract (Granada). 2013 4;11(2):71–80. DOI: 10.4321/s1886-36552013000200003. 24155853 PMC3798172

[25] Naranjo CA, Busto U, Sellers EM, Sandor P, Ruiz I, Roberts EA, Janecek E, Domecq C, Greenblatt DJ A method for estimating the probability of adverse drug reactions. Clin Pharmacol Ther. 1981 8;30(2):239–45. DOI: 10.1038/clpt.1981.154. 7249508

[26] Arimone Y, Bégaud B, Miremont-Salamé G, Fourrier-Réglat A, Moore N, Molimard M, Haramburu F Agreement of expert judgement in causality assessment of adverse drug reactions. Eur J Clin Pharmacol. 2005 5;61(3):169–73. DOI: 10.1007/s00228-004-0869-2. 15827761

[27] Arimone Y, Miremont-Salamé G, Haramburu F, Molimard M, Moore N, Fourrier-Réglat A, Bégaud B Inter-expert agreement of seven criteria in causality assessment of adverse drug reactions. Br J Clin Pharmacol. 2007 10;64(4):482–8. DOI: 10.1111/j.1365-2125.2007.02937.x. 17711539 PMC2048553

[28] Behera SK, Das S, Xavier AS, Velupula S, Sandhiya S Comparison of different methods for causality assessment of adverse drug reactions. Int J Clin Pharm. 2018 8;40(4):903–10. DOI: 10.1007/s11096-018-0694-9. 30051231

[29] TangiisuranB, AuyeungV, CheekL, RajkumarC, DaviesG Interrater reliability of the assessment of adverse drug reactions in the hospitalised elderly. J Nutr Health Aging. 2013;17(8):700–5.2409702510.1007/s12603-013-0011-1

[30] Sinayev A, Peters E, Tusler M, Fraenkel L Presenting numeric information with percentages and descriptive risk labels: a randomized trial. Med Decis Making. 2015 11;35(8):937–47. DOI: 10.1177/0272989X15584922. 25952743 PMC4592369

[31] Peters E, Hart S, Fraenkel L Informing patients: the influence of numeracy, framing, and format of side effect information on risk perceptions. Med Decis Making. 2011 May-Jun;31(3):432–6. DOI: 10.1177/0272989X10391672. 21191122

[32] Lipkus IM Numeric, verbal, and visual formats of conveying health risks: suggested best practices and future recommendations. Med Decis Making. 2007 Sep-Oct;27(5):696–713. DOI: 10.1177/0272989X07307271. 17873259

[33] US Food and Drug Administration. Part III: drugs and foods under the 1938 act and its amendments [Internet] The Administration [cited 28 Jan 2020]. <https://www.fda.gov/about-fda/fdas-evolving-regulatory-powers/part-iii-drugs-and-foods-under-1938-act-and-its-amendments>.

